# Curbing and preventing psychiatric disorders through healthier eating and sleeping habits

**DOI:** 10.1016/j.amsu.2022.104614

**Published:** 2022-09-09

**Authors:** Muhammad Saad Tahir, Mohammad Ebad Ur Rehman, Faizan Fazal, Hania Murtaza, Ayesha Noor, Asmara Kamran, Usama Tanveer, Haris Mustafa

**Affiliations:** Department of Medicine, Rawalpindi Medical University, Block E Satellite Town, Rawalpindi, Pakistan

**Keywords:** Psychiatric disorders, Preventing

WHO defines health as being subdivided into 3 important components: Mental, social and physical health. Health is not only the mere absence of a disease rather it encompasses wellbeing in all 3 subcategories of health [1]. In the present day and age we have come to believe that mental health is an important component of the over all health. Mental health instills in us the everyday abilities such as stress coping, realizing our abilities, to learn and work efficiently, and to contribute positively towards our community [2].

Mental health is a broad health concept that depends not only on any single factor. It is governed and affected by some important aspects such as gender, race, age and socioeconomic status [3]. A psychiatric disorder is defined in the ICD 10 as “a clinically recognizable set of symptoms or behaviours associated in most cases with distress and with interference with personal functions” [4].

As far as the treatment of psychiatric disorders is concerned, these disorders are traditionally treated with pharmacological therapy^%^ and non-pharmacological therapy such as cognitive behavior therapy(CBT) [5]. Although these treatments have proven to be effective in treating psychiatric disorders, but these are costly and in many cases inaccessible specifically to the lower socioeconomic class people which makeup bunk of the total population of developing countries. It has been shown that the prevalence of depression is most amongst the lower socioeconomic class [6]. A question then arises that, “are there some other cost effective alternative ways in which psychiatric disorders can be prevented and/or treated so that the deprived classes of society can benefit and thus have a better and healthier mental health?"

Fortunately, the answer to the above poised question is yes. Healthy diet and better sleep habits have been shown to improve the mental health status of people in various studies conducted worldwide. A healthy and a balanced diet in children and adults has proven to produce people with better mental health with there being evidence for the reverse as well [7]. Diet is now even regarded as a modifiable risk factor in a number of psychiatric disorders such as anxiety and depression. Diet is now thought to be involved in the pathogenesis of these psychiatric disorders [8]. Microbiome of gut and diet have shown to influence the mood status and thus both factors have been shown to be involved in the psychiatric disorders [9]. In a prospective study done on adolescents, it was concluded that improvements in diet can lead to improvement in the mental health status [10]. This is of utmost significance because the root of pathogenesis of many mental disorders is in the period of adolescence [10]. Thus it can safely said that eating a balanced, healthy diet can play a major role in prevention and control of some psychiatric disorders.

The second cost effective alternative to improve mental health is good sleep. A randomized controlled trial showed that in patients having symptoms of hallucinations, paranoia and associated insomnia, treating the insomnia also treated much of the hallucinations and paranoia [11]. This signifies how important sleep can be in the prevention and cure for psychiatric disorders. Other studies have also shown the role of sleep in improving mental health in general [12]. And sleep quality for most people can be easily improved by simple habit changes that costs us nothing financially. These habits involve having a consistent bedtime, a dark comfortable room to sleep in, avoiding looking at screens before bedtime, getting regular exercise and avoiding large meals and caffeine before sleeping.

Thus it can be seen that it is possible to improve the mental health of people and prevent as well as help cure psychiatric disorders using inexpensive ways like improving diet and sleep quality. What is needed is to increase the level of awareness among the masses so that our societies can reap the benefits of better mental health.

## Ethical approval

Not applicable.

## Sources of funding

None.

## Author contributions

Mohammad Ebad Ur Rehman: Study conception, write-up, critical review and approval of the final version.

Muhammad Saad Tahir:Study conception, critical review and approval of the final version.

Faizan Fazal: Study conception, write-up, critical review and approval of the final version.

Hania Murtaza: Study conception, critical review and approval of the final version.

Aisha noor: Study conception, write-up, critical review and approval of the final version.

Asmara Kamran: Study conception, write-up, critical review and approval of the final version.

Usama Tanveer: Study conception, write-up, critical review and approval of the final version.

Haris Mustafa: Study conception, write-up, critical review and approval of the final version.

## Registration of research studies

1 Name of the registry: Not applicable
2 Unique Identifying number or registration ID: Not applicable
3 Hyperlink to your specific registration (must be publicly accessible and will be checked): Not applicable

## Guarantor

Mohammad Ebad Ur Rehman.

Faizan Fazal.

## Consent

Not applicable.

## Declaration of competing interest

The authors have no conflicts of interest.
